# Ten-Eleven Translocation-2 (Tet2) Is Involved in Myogenic Differentiation of Skeletal Myoblast Cells *in Vitro*

**DOI:** 10.1038/srep43539

**Published:** 2017-03-08

**Authors:** Xia Zhong, Qian-Qian Wang, Jian-Wei Li, Yu-Mei Zhang, Xiao-Rong An, Jian Hou

**Affiliations:** 1 State Key Laboratory of Agrobiotechnology and College of Biological Science, China Agricultural University, Beijing, China

## Abstract

Muscle cell differentiation is a complex process that is principally governed by related myogenic regulatory factors (MRFs). DNA methylation is considered to play an important role on the expression of MRF genes and on muscle cell differentiation. However, the roles of enzymes specifically in myogenesis are not fully understood. Here, we demonstrate that Tet2, a ten-eleven translocation (Tet) methylcytosine dioxygenase, exerts a role during skeletal myoblast differentiation. By using an immunostaining method, we found that the levels of 5-hydroxymethylcytosine (5-hmC) were much higher in differentiated myotubes than in undifferentiated C2C12 myoblasts. Both Tet1 and Tet2 expression were upregulated after differentiation induction of C2C12 myoblasts. Knockdown of Tet2, but not Tet1, significantly reduced the expression of myogenin as well as Myf6 and myomaker, and impaired myoblast differentiation. DNA demethylation of myogenin and myomaker promoters was negatively influenced by Tet2 knockdown as detected by bisulfite sequencing analysis. Furthermore, although vitamin C could promote genomic 5hmC generation, myogenic gene expression and myoblast differentiation, its effect was significantly attenuated by Tet2 knockdown. Taken together, these results indicate that Tet2 is involved in myoblast differentiation through promoting DNA demethylation and myogenic gene expression.

Muscle differentiation is a highly conserved process that occurs through the activation of quiescent satellite cells whose progeny proliferates, differentiates, and fuses to generate new myofibers. The course of skeletal myogenesis is precisely orchestrated by the myogenic regulatory factors (MRFs), such as MyoD, Myf5, myogenin, and Myf6 (also known as MRF4)^1^,^2^. Myf5 and MyoD are primary MRF proteins expressed in myoblast stage and are essential for skeletal muscle lineage determination, whereas myogenin and Myf6 are expressed upon myoblast differentiation into myotubes and probably collaborated with MyoD, control terminal muscle differentiation^3^,^4^,^5^,^6^,^7^,^8^,^9^. These myogenic factors cooperate with each other to regulate myogenic progress and promote the expression of some important genes for muscle cell function, such as myosin heavy chain (MyHC) and the recently discovered myomaker (also called Tmem8c)^10^,^11^.

In mammals, DNA cytosine methylation is one of the key epigenetic marks and has been suggested to play an important role on muscle development^12^. The initial correlation between DNA methylation and myogenesis is the observation that C3H10T1/2 embryonic fibroblasts were transformed into muscle cells by treatment with the DNA methyltransferase(DNMT) inhibitor 5-azacytidine^13^. This correlation has been further reinforced by the findings that promoters of MRF genes, *MyoD* and *myogenin*, were demethylated during myogenesis^14^,^15^,^16^. In particular, demethylation of *myogenin* promoter is highly correlated with transcriptional activation of this gene and with muscle terminal differentiation^16^,^17^,^18^. Furthermore, treatment of C2C12 myoblast cells with 5-azacytidine upregulated the expression of muscle related genes and enhanced the myotube maturation^19^. Although these studies have provided many insights of DNA methylation associated with myogenesis, the precise mechanism regulating demethylation during muscle differentiation *in vivo* is still poorly understood.

In recent years, dioxygenases of the ten-eleven translocation (Tet) family have been discovered to have the capacity of catalyzing the conversion of 5-methylcytosine (5mC) to 5-hydroxymethylcytosine (5hmC)^20^,^21^. Subsequent studies demonstrate that Tet proteins can further oxidize the 5hmC to 5-formylcytosine (5fC) and 5-carboxylcytosine (5caC), which can be excised by thymine-DNA glycosylase (TDG) to regenerate unmodified cytosines^22^,^23^,^24^. Tet protein-initiated oxidation of 5mC provides a solid pathway for active demethylation *in vivo* and has been shown to be associated with various biological and pathological processes during mammalian development^25^,^26^. However, it remains unknown whether or how Tet proteins act on skeletal myogenesis, although one recent report has found that the transcripts of two TET members, *TET1* and *TET2*, were strongly upregulated in human skeletal myoblasts and myotubes^27^.

In this study, we have revealed, in murine C2C12 myoblasts, a well-established model system for skeletal myogenesis, that Tet2 exerts a role on skeletal muscle differentiation. We find that 5hmC is enriched in differentiated myotubes and Tet2 contributes to this event. Tet2-induced genome-wide 5hmC generation or specific gene demethylation is crucial for myoblast differentiation. We also show that vitamin C can promote myogenic differentiation, but its effect is dependent on Tet2-involved pathway. Our results provide evidence supporting a Tet protein-mediated active demethylation mechanism that regulates skeletal myogenesis.

## Results

### 5-hmC and Tet expression are upregulated during C2C12 differentiation

**Figure 1:**
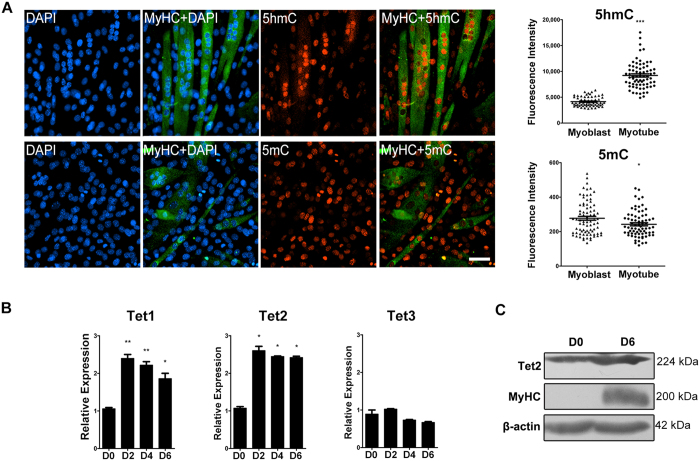
Tet expression and 5hmC levels are increased during myoblast differentiation. (**A**) *Left*: immunofluorescence of 5hmC (top row) or 5mC (bottom row) in C2C12 cells after differentiation induction for 6 d. Differentiated myotubes were marked by immunostaining for myosin heavy chain (MyHC) and nuclei were stained with DAPI. Scale bar, 100 μm; *Right*: quantification of 5hmC and 5mC immunofluorescence intensities in nuclei of differentiated and undifferentiated cells. Data are presented as means ± SEM. *p < 0.05; ***p < 0.001. (**B**) qRT-PCR analysis for the expression of Tet family genes in C2C12 cells at differentiation day 0, 2, 4 and 6. Gapdh was used as an internal control. Data are presented as means ± SEM (n = 3). Asterisks above columns represent significant difference among groups (p < 0.05). (**C**) Western blot analysis of Tet2 protein in C2C12 cells at differentiation day 0 and 6. MyHC is a marker of muscle differentiation and β-actin was used as an internal control. The expected Tet2 band (NW: 224 kDa) is shown. Full-length blots are presented in Supplementary Figure S6.

To determine whether DNA demethylation occurs during myoblast differentiation, we detected the levels of 5hmC and 5mC in C2C12 cells 6 d after differentiation induction, by using immunostaining with antibodies against 5hmC and 5mC, respectively. Assessment by immunostaining for MyHC, a differentiation marker of skeletal muscle cells, indicated that approximately 30% of the cells were differentiated and many of them formed multinucleated myotubes. As shown in Fig. 1A, the nucleus in differentiated myotubes contained much higher levels of 5hmC than that in undifferentiated mononuclear cells, while 5mC levels were generally lower in differentiated cells as compared to the undifferentiated cells. Indeed, we observed gradual increase of 5hmC in cells with differentiation (Supplementary Figure S1A). These results suggest that the cells may have initiated demethylation during C2C12 differentiation.

As Tet proteins are the only known enzymes that convert 5mC to 5hmC, we examined the expression of all three members of Tet protein family during C2C12 differentiation. qRT-PCR analysis showed that both *Tet1* and *Tet2* transcripts were dramatically increased in the cells after differentiation induction for 2 d, while *Tet3* expression was not significantly altered by differentiation and remained a very low level (Fig. 1B). In particular, *Tet2* maintained a high level of expression during subsequent differentiation. Western blot analysis further confirmed the upregulation of Tet2 expression in differentiated cells (Fig. 1C). Immunostaining indicated that Tet2 protein was localized, in punctate patterns, in the nuclei of myoblasts and differentiated myotubes (Supplementary Figure S1B). These results suggest a possible role of Tet2 (and/or Tet1) on myoblast differentiation.

### Knockdown of Tet2 decreases the expression of myoblast differentiation-associated genes

**Figure 2:**
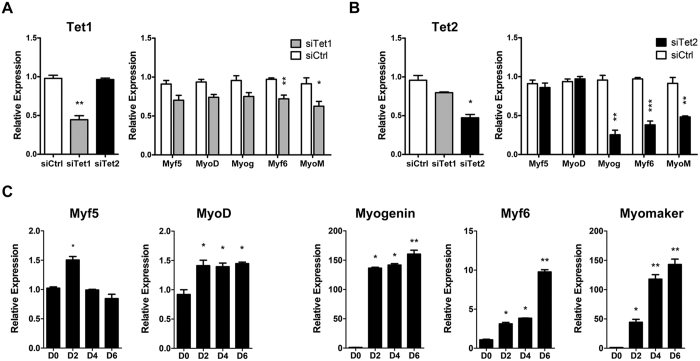
Knockdown of Tet2 decreases the expression of myoblast differentiation-associated genes. (**A**) and (**B**) Knockdown of Tet expression. C2C12 cells were transfected with Tet1 or Tet2 siRNAs (siTet) or control siRNA (siCtrl). Forty-eight hours after transfection, mRNA levels of Tet1, Tet2 and muscle-specific genes were analyzed by qRT-PCR. Tet1 or Tet2 mRNA levels were decreased by more than 50% by their specific siRNAs when compared to that by control siRNA (left row in A or B). The expression of several muscle-specific genes was downregulated in Tet1 or Tet2 knockdown cells (right row in A or B). Note that Tet2 knockdown led to dramatic decrease of myogenin (Myog), Myf6 and myomaker (MyoM) expression. *p < 0.05; **p < 0.01; ***p < 0.001 as compared with C2C12 transfected by control siRNA. (**C**) The expression levels of muscle-specific genes during C2C12 differentiation. Gapdh was used as an internal control. Data are presented as means ± SEM (n = 3). Asterisks above columns represent significant difference among the groups (p < 0.05).

To investigate the roles of Tet1 and Tet2 on myoblast differentiation, we knocked down their expression in C2C12 by using short interfering RNAs (siRNA). When transfected into cells, these siRNAs specifically decreased mRNA levels of *Tet1* or *Tet2* to below 50% as compared with the control siRNA (Fig. 2A). We then examined the influence of Tet1- or Tet2-knockdown on expression of myogenesis-associated genes, including *Myf5, MyoD, myogenin, Myf6* and *myomaker*. As shown in Fig. 2A, Tet1 knockdown only led to moderate decrease of *Myf6* and *myomaker* transcripts and had no significant influence on *Myf5, MyoD* and *myogenin. MyoD* or *Myf5* expression was also not changed by knockdown of Tet2, but the transcripts of *myogenin, Myf6* and *myomaker* were dramatically downregulated in Tet2 knockdown cells (Fig. 2B). In normal C2C12 cells during differentiation, we observed a sharp rise of *myogenin* expression after 2 d of differentiation induction (Fig. 2C). In addition, *Myf6* and *myomaker* expression were also gradually increased with C2C12 differentiation. This result supports the concept that myogenin, as well as Myf6 and myomaker, exert critical roles during skeletal muscle terminal differentiation^4^,^5^,^6^,^10^,^11^. As Tet2 knockdown significantly downregulated the expression of myoblast differentiation-associated genes, we speculated that Tet2 may be involved in myoblast differentiation through promoting the expression of these genes.

### Tet2 knockdown impairs myoblast differentiation

**Figure 3:**
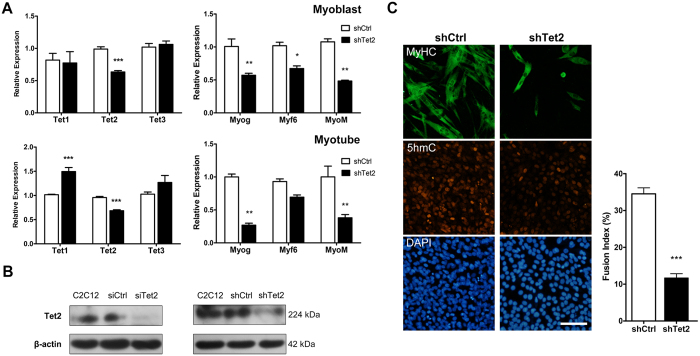
Tet2 knockdown decreases myoblast differentiation. (**A**) Knockdown of Tet2 by using shRNA. *Upper*: C2C12 cells were transfected with Tet2-specific shRNA plasmid (shTet2) or a control shRNA plasmid (shCtrl) and then selected with puromycin. The mRNA levels of Tet family genes or myoblast differentiation-associated genes in puromycin-selected cells were detected by qRT-PCR. *Lower*: the cells were induced to differentiation and the mRNA levels of Tets or myoblast differentiation-associated genes were detected 4 d after differentiation induction. Myog, myogenin; MyoM, myomaker. (**B**) Western blot confirmation of Tet2 protein decline in shRNA- or siRNA-mediated Tet2 knockdown cells. β-actin was used as an internal control. The expected Tet2 band (NW: 224 kDa) is shown. Full-length blots are presented in Supplementary Figure S6. (**C**) Evaluation of myotube formation of Tet2 knockdown C2C12 cells. Myotubes were visualized by immunostaining for MyHC 6 d after differentiation induction (left). Nuclei were stained with DAPI. Scale bar, 100 μm. The fusion index was calculated as the ratio of the number of nuclei in MyHC-positive cells to the total number of nuclei present in the observation field. The quantitative analysis revealed a significant decrease in the fusion index of shTet2 C2C12 as compared with shCtrl cells (right). Data are presented as means ± SEM (n = 3). *p < 0.05; **p < 0.01; ***p < 0.001.

Due to the inability of siRNAs to silence *Tet2* expression for long time, we used short hairpin RNA (shRNA)-expressing plasmid to achieve prolonged silencing of *Tet2* expression. As shown in Fig. 3A, the *Tet2* transcripts were significantly reduced in cells transfected with the *Tet2* shRNA plasmid (shTet2) as compared with the cells transfected with a control shRNA plasmid (shCtrl). Tet2 protein was also decreased and was less localized in shTet2-induced knockdown cells in comparison with the control cells (Fig. 3B and Supplementary Figure S2). Similar to siRNA-mediated Tet2 knockdown (Fig. 2B), knockdown by Tet2 shRNA significantly reduced the transcripts of *myogenin, Myf6* and *myomaker* in C2C12 cells (Fig. 3A). After the cells were induced to differentiate, the expression of these myoblast differentiation-associated genes was persistently inhibited by Tet2 knockdown (Fig. 3A). Interestingly, although Tet2 knockdown did not affect the expression of *Tet1* in myoblasts, it led to significant increase of *Tet1* expression in differentiated cells. We then examined the influence of Tet2 knockdown on C2C12 differentiation. Immunostaining for MyHC indicated that the percentage of nuclei fused into myotubes was reduced from 34.5% in control cells to 11.7% in Tet2 knockdown cells (Fig. 3C). These results suggest that Tet2 has a role on myoblast differentiation.

### Tet2 is involved in demethylation of myogenin and myomaker promoters

**Figure 4:**
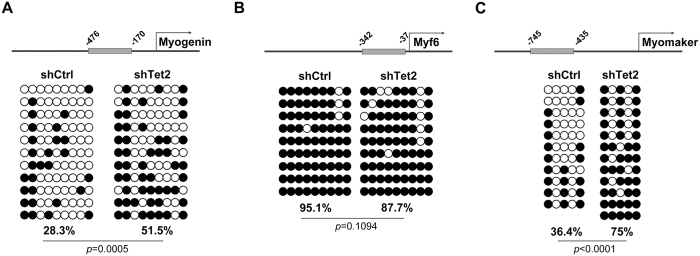
Promoter methylation status of myoblast differentiation-associated genes in Tet2 knockdown C2C12. C2C12 cells transfected with Tet2 shRNA (shTet2) or control shRNA (shCtrl) were cultured in growth medium. Methylation status of promoters of myogenin (**A**), Myf6 (**B**) or myomaker (**C**) was analyzed by using bisulfite sequencing. Numbers with a minus sign indicate the position of CpG relative to the transcription starting site. Each row represents an individual clone sequenced; black and white circles represent methylated and unmethylated CpGs, respectively. Percentage of methylation indicates the proportion of methylated CpG sites relative to the whole CpG sites examined.

To determine the association between gene expression and Tet2-induced demethylation, we examined the methylation status of gene promoters by using bisulfite sequencing. A total of 9 CpG sites located in the region from −401 to −176 bp of *myogenin* promoter were analyzed. As shown in Fig. 4A, the methylation level of *myogenin* promoter was 28.3% in C2C12 cells transfected with control shRNA, but it was increased up to 51.1% in Tet2 knockdown cells. For *myomaker*, 5 CpG sites in the promoter region from −745 to −435 bp were examined. Similar to that in *myogenin*, Tet2 knockdown significantly increased *myomaker* promoter methylation, from 36.4% in control cells to 75% in Tet2 knockdown cells (Fig. 4C). However, 9 CpG sites tested for *Myf6* promoter remained highly methylated in both Tet2 knockdown and control cells (Fig. 4B). These results suggest that Tet2 is involved in DNA demethylation of specific genes, and such demethylation may be correlated with the expression of relevant genes, such as *myogenin* and *myomaker*.

### Knockdown of Tet2 decreases the effects of vitamin C in myoblast differentiation

**Figure 5:**
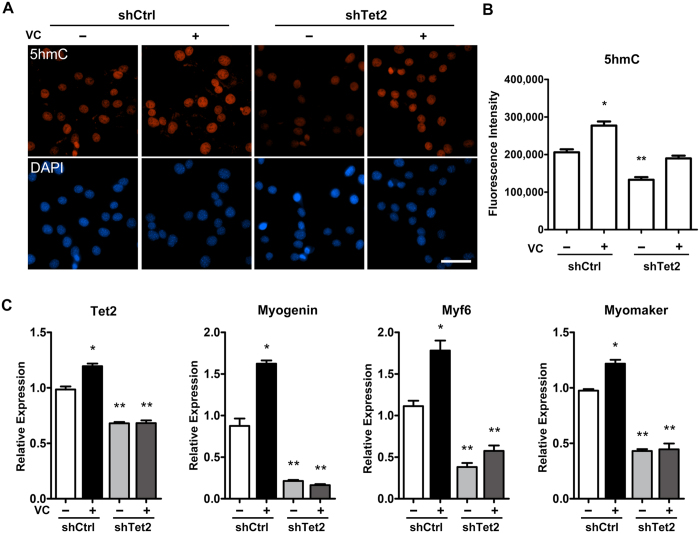
Knockdown of Tet2 decreases the ability of vitamin C to enhance 5hmC formation and to promote the expression of myoblast differentiation-associated genes. C2C12 cells transfected with Tet2 shRNA (shTet2) or control shRNA (shCtrl) were cultured in growth medium with or without vitamin C (VC; 500 μM). (**A**) Immunostaining for 5hmC in shTet2 vs shCtrl C2C12 cells with or without VC treatment. Nuclei were stained with DAPI. Scale bar, 50 μm. (**B**) Quantification of fluorescence intensities of 5hmC. The quantitative analysis revealed that Tet2 knockdown decreased the ability of VC to enhance 5hmC formation. (**C**) qRT-PCR analysis for the expression of myogenin, Myf6 and myomaker in shTet2 vs shCtrl C2C12 cells with or without VC treatment. Gapdh was used as an internal control. Data are presented as means ± SEM (n = 3). Asterisks above columns represent significant difference among groups (p < 0.05).

It was reported recently that vitamin C can enhance the 5hmC generation through promoting Tet activity^28^,^29^,^30^,^31^. We tested the effect of vitamin C in C2C12 cells and demonstrated that vitamin C could promote 5hmC generation, myogenic gene expression and myoblast differentiation (Supplementary Figures S2 and 3). Based on these observations, we addressed whether vitamin C interplays with Tet2. C2C12 cells were transfected with shTet2 plasmid to knockdown the expression of Tet2. As expected, transfection with shTet2 resulted in significant 5hmC decrease when compared to the transfection with shCtrl plasmid (referred as to non-knockdown thereafter) (Fig. 5A and B), confirming that Tet2 is crucial for genome-wide 5hmC generation. Vitamin C induced the 5hmC level increase in Tet2 knockdown cells, but this effect was not comparable to its effect in non-knockdown cells (Fig. 5A and B). In Tet2 knockdown cells, the expression of *myogenin, Myf6* and *myomaker* was dramatically decreased, even in the presence of vitamin C, while in non-knockdown cells, the expression of these genes was significantly increased by vitamin C (Fig. 5C). These results suggest that knockdown of Tet2 had impaired the effect of vitamin C on promoting the generation of 5hmC and the expression of myoblast differentiation-associated genes.

**Figure 6:**
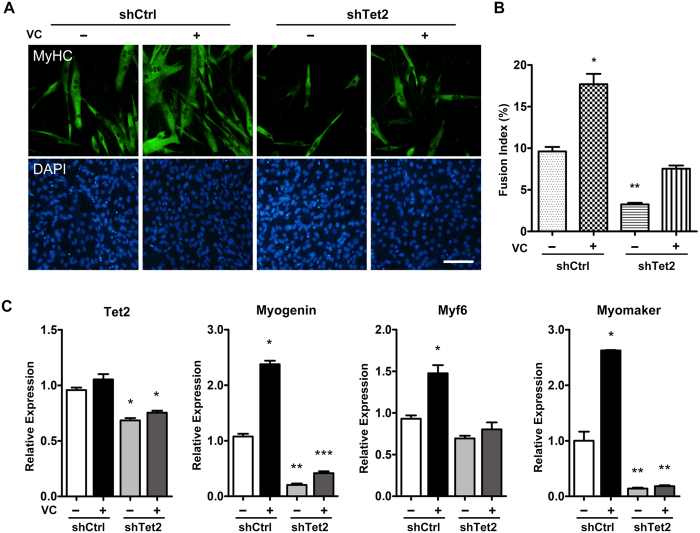
Knockdown of Tet2 decreases the ability of vitamin C to promote myoblast differentiation. C2C12 cells transfected with Tet2 shRNA (shTet2) or control shRNA (shCtrl) were subdivided into two experimental groups: non-vitamin C treatment group (indicated as VC−), in which VC was absent in both growth medium and differentiation medium, and VC-treatment group (indicated as VC+), in which the cells were first cultured for 48 h in growth medium containing 500 μM VC, and then were shifted to differentiated medium containing 500 μM VC. (**A**) Evaluation of myoblast differentiation in different treatment groups. Cells after 6 d of differentiation induction were immunostained with anti-MyHC antibodies to mark the myotubes. Nuclei were stained with DAPI. Scale bar, 100 μm. (**B**) Quantification analysis for differentiation efficiency in different treatment groups. The fusion index was calculated as the ratio of the number of nuclei in MyHC-positive cells to the total number of nuclei present in the observation field. (**C**) qRT-PCR analysis for the expression of myogenin, Myf6 and myomaker in C2C12 cells after 4 d of differentiation induction in different treatment groups. Gapdh was used as an internal control. Data are presented as means ± SEM (n = 3). Asterisks above columns represent significant difference among groups (p < 0.05).

We next investigated the relationship between vitamin C and Tet2 in differentiated C2C12 cells. Tet2 knockdown cells and non-knockdown cells were cultured sequentially in growth medium (GM) and differentiation medium (DM), both supplemented with or without vitamin C. The differentiation was evaluated after 6 d of culture in DM. As shown in Fig. 6A and B, Tet2 knockdown dramatically decreased the efficiency of myotube formation, and vitamin C significantly enhanced myotube formation in both Tet2 knockdown cells and non-knockdown cells. However, the enhancement of myotube formation by vitamin C in Tet2 knockdown cells was attenuated as compared to that in non-knockdown cells, suggesting that Tet2 knockdown had impaired the effect of vitamin C on promoting myoblast differentiation. Furthermore, regardless of the presence of vitamin C, the expression of *myogenin* and *myomaker* was persistently inhibited in Tet2 knockdown cells after differentiation induction (Fig. 6C), which was similar to the situation in non-induction C2C12 cells (Fig. 5C). However, the *Myf6* expression in Tet2 knockdown cells was not increased by vitamin C.

## Discussion

In this study, we have suggested that Tet2 plays a role on differentiation of skeletal muscle cells. The recently discovered Tet family proteins have been shown to mediate DNA demethylation process by oxidizing 5mC to 5hmC. Tet proteins have important roles in epigenetic reprogramming in early embryos, primordial germ cells and embryonic stem cells^25^,^26^. For Tet2, null mutation studies have supported a role of Tet2 in haematopoiesis^32^,^33^,^34^,^35^, and more recently, Tet2 functions have been implicated in smooth and cardiac muscle^36^,^37^. Our study, for the first time, provides evidence that Tet2 is involved in skeletal myogenesis.

It is now known that 5hmC is a derivative from oxidized 5mC by Tet proteins and is a key intermediate in active demethylation pathway. Although 5hmC remains extremely low levels in most tissues, it is abundant in some cell types, for example mouse Purkinjie neurons and embryonic stem cells^21^,^38^. In the present study, we found that 5hmC was highly correlated with skeletal muscle differentiation in that the nuclei of differentiated myotubes contained much more content of 5hmC than those undifferentiated myoblast cells. Recent studies described a clear loss of genomic DNA methylation during skeletal muscle terminal differentiation^27^,^39^. Enzymatic assays for genomic 5hmC showed that 5hmC was more enriched in mature skeletal muscle as compared with myogenic progenitor cells including both myoblasts and myotubes^27^. In our study, we adopted immunostaining method to separately mark 5mC and 5hmC, and at the same time, to discriminate between differentiated myotubes and undifferentiated cells in the same cultures. Our results indicate a remarkable increase in DNA hydroxymethylation in myotubes as compared with myoblasts and suggest a more modest decrease in global DNA methylation during differentiation of myoblasts to myotubes.

Accumulating of 5hmC upon myoblast differentiation suggests that active demethylation probably occurs during this process. We found that the expression of *Tet1* and *Tet2*, but not *Tet3*, was dramatically increased in C2C12 cells after induction of differentiation. Using microarray profiling analysis, Tsumagari *et al*. recently showed that skeletal muscle lineage cells, including both myoblasts and myotubes, contained much more *Tet1* and *Tet2* transcripts than most non-muscle cell strains^27^. Our study has further explored this observation in that induced differentiation led to further upregulation of *Tet1* and *Tet2* expression in C2C12 when compared with that prior to differentiation. This result suggests that Tet proteins are probably responsible for the formation of 5hmC in myotubes and may be involved in myoblast differentiation.

Skeletal muscle cell differentiation is principally regulated by several muscle-specific proteins, including MyoD, Myf5, myogenin, Myf6 and the recently discovered myomaker. It is shown that myogenin, as well as Myf6 and myomaker, are required for myoblast differentiation into myotubes^4^,^5^,^6^,^10^,^11^. Consistent with previous reports, we observed marked increase of the expression of *myogenin, Myf6* and *myomaker* in C2C12 cells after differentiation induction. We have further demonstrated the role of Tet2 on regulating the expression of these genes. Knockdown of Tet2, but not Tet1, resulted in dramatic reduction of *myogenin* expression. Myogenin is considered to be a differentiation master that is essential for myoblast differentiation^5^,^6^,^40^. The expression of *Myf6* and *myomaker*, the other two myogenic differentiation-associated genes, was also greatly downregulated by Tet2 knockdown. Furthermore, knockdown of Tet2 alone severely impaired the myoblast differentiation. While Tet1 is highly expressed in Tet2 knockdown cells, it seemed to have no or only have a partial compensation for the silence of Tet2, because the phenotype of knockdown cells was not completely rescued. Taken together these results, we can conclude that Tet2 has a critical role on *in vitro* myoblast differentiation through promoting the expression of differentiation-associated genes.

There are several evidences supporting the positive correlation between DNA demethylation and *myogenin* expression^15^,^16^,^17^,^18^. In particular, previous observations of the fast demethylation of *myogenin* 5′ flanking region upon differentiation induction have suggested that an active demethylation mechanism may exist in myoblast differentiation^16^,^17^. However, the enzymes involved in this process are poorly understood. In this study, using bisulfite sequencing analysis, we showed that knockdown of Tet2 in C2C12 cells significantly increased the methylation level of *myogenin* promoter sites. This result indicates that Tet2 has a function on *myogenin* promoter demethylation. Interestingly, Tet2 knockdown also led to increased methylation in *myomaker* promoter. However, due to the small number of CpG sites existing in *myomaker* promoter region, the correlation between DNA methylation and gene expression for *myomaker* still needs further investigation. In contrast to *myogenin* and *myomaker, Myf6* promoter remained hypermethylation in both Tet2 knockdown cells and control cells. Thus, *Myf6* expression might not be correlated with DNA methylation, as suggested by a previous study^41^. Downregulated expression of *Myf6*, as well as *myomaker*, in Tet2 knockdown cells, may be due to the reduction of *myogenin* expression, as myogenin has been shown to be a potential upstream activator for *Myf6* and *myomaker* transcription^10^,^11^,^42^. In addition to *myogenin*, other factors might also regulate the expression of *Myf6*. For example, it is shown that MEF2 plays a role in activating the *Myf6* promoter^42^.

Bisulfite sequencing analysis indicate that Tet2 induces the loss of both 5mC and 5hmC modifications in some specific gene sites, at least for the *myogenin* gene. However, the stably maintained 5hmC in differentiated myotubes implies that no further or complete demethylation happens in genome-global levels, since 5hmC is generally considered to be an intermediate in DNA demethylation. Two pathways, either “active” or “passive”, have been proposed to mediate complete demethylation following 5hmC oxidation^25^,^26^. One possible explanation for the accumulation of 5hmC in myotubes is that DNA replication-dependent passive demethylation is blocked due to the mitotic arrest of fused nuclei in myotubes. Complete demethylation of some subtle gene sites might occur through an active pathway, for example, thymine DNA glycosylase (TDG)-mediated base excision repair^22^,^24^. In addition, Dnmt family proteins may also have functions in DNA demethylation in the myogenic process, as we observed dynamic changes of Dnmt expression during myoblast differentiation (Supplementary Figure S5). Interestingly, Tet2 knockdown led to upregulated expression of both *Dnmt1* and *Dnmt3a*, although the letter two displayed differential expression with the myoblast differentiation (Supplementary Figure S5). These observations suggest a link between Tet and Dnmt enzymes in regulating the demethylation of muscle-specific genes. Future investigation is required to address how these methyltransferases and demethylases coordinate the demethylation process during myoblast differentiation.

**Figure 7:**
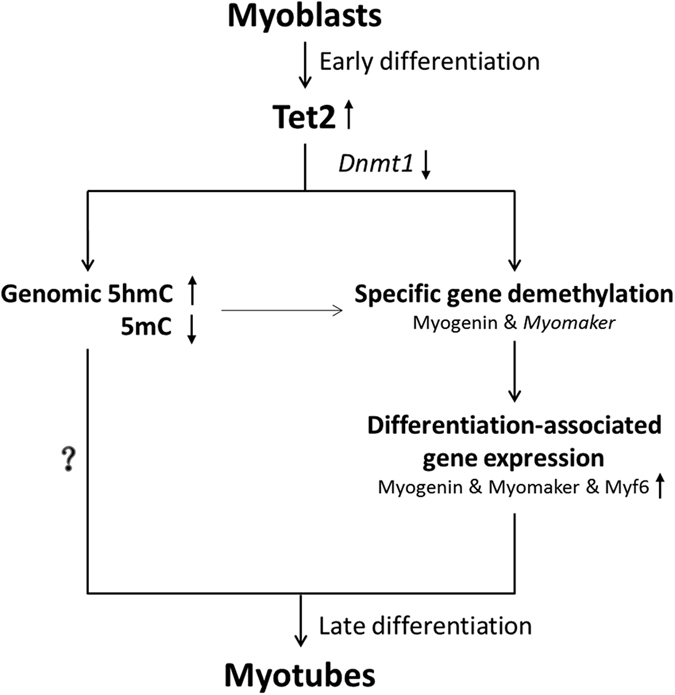
Proposed model of Tet2-involved myoblast differentiation. Tet2 expression is upregulated in myoblasts after differentiation induction. Tet2 directly induces hydroxymethylation and subsequent demethylation of differentiation-associated genes (eg, myogenin and myomaker). Besides, downregulation of Dnmt1 may lead to a passive demethylation in this process. Demethylation results in activation and expression of the differentiation-associated genes, which drives myotube formation.

It is also possible that 5hmC *per se* might directly involve epigenetic regulation of gene functions^43^. In this regard, Terragni *et al*. recently suggested that high levels of 5hmC at Notch signaling genes in skeletal muscle may help fine-turn expression of the genes that is required for various muscle-specific activities, for example, regeneration of injured skeletal muscle^44^. The biological significance of 5hmC abundant in differentiated muscle cells needs to be elucidated in future studies. Based on our findings, we propose that Tet2 induces hydroxymethylation and promoter demethylation of some specific genes (eg. *Myogenin*), leading to the activation and expression of myoblast differentiation-associated genes, which consequently promotes the differentiation of myoblast into myotubes (Fig. 7).

Early studies have reported that vitamin C can promote muscle differentiation^45^. It was discovered until recently that vitamin C can act as a regulator of Tet activity to enhance the generation of 5hmC^28^,^29^,^30^,^31^. In this study, we have provided evidence linking these two vitamin C-mediated biological processes in myogenic cells. Vitamin C can promote the 5hmC generation, the expression of myogenic genes, and the differentiation of C2C12 myoblasts. The similar roles between vitamin C and Tet2 in regulating 5hmC generation and myoblast differentiation promoted us to speculate that the effect of vitamin C is dependent on Tet2-involved pathway. To this end, we examined the function of vitamin C in Tet2 knockdown cells. We showed that knockdown of Tet2 dramatically decreased the efficacy of vitamin C in enhancing 5hmC generation, *myogenin/myomaker* expression and myotube formation. These results demonstrate that Tet2 is implicated in vitamin C-regulated myogenic process. However, we found that vitamin C could partially rescue some phenotypes of Tet2 knockdown cells, such as slightly increasing 5hmC generation and myotube formation. This might be due to that vitamin C had enhanced the activity of the residual Tet2 protein that resulted from incomplete RNAi. Also possibly, other pathways in association with vitamin C might contribute, to some extents, to the myogenic process. Nevertheless, the fact that the effect of vitamin C in C2C12 cells was severely impaired by Tet2 knockdown support the role of Tet2 in myoblast differentiation.

In summary, our study demonstrates that genome-wide hydroxymethylation occurs during skeletal myogenic differentiation and Tet proteins act as important participators in this process. Tet2-mediated gene-specific demethylation is crucial for myoblast differentiation by regulating relevant gene expression and is also involved in regulatory pathway induced by other agents like vitamin C. Our study provides new insights for understanding the mechanism of skeletal muscle differentiation.

## Methods

### Culture, differentiation, and treatment of mouse C2C12 skeletal myoblast

Mouse skeletal muscle myoblasts C2C12 cells were purchased from CAS typical Culture Collections Committee cell library. C2C12 cells were cultured in growth medium (GM), Dulbecco’s Modified Eagle’s Medium (DMEM, Invitrogen) containing 10% (v/v) fetal bovine serum (FBS; HyClone), 100 units/ml penicillin and 100 μg/ml streptomycin sulfate (Invitrogen) at 37 °C in 5% CO2. To induce differentiation, when reached 90–100% of confluence, cells were shifted to differentiation medium (DM), DMEM supplemented with 2% (v/v) horse serum (HyClone) and antibiotics. Vitamin C (L-Ascorbic acid 2-phosphate, Sigma) was added into GM or DM according to the experimental design. The day of shift to DM was indicated as day 0 of differentiation.

### RNA extraction and quantitative PCR analysis

Total RNA from C2C12 cells was isolated using the TRNzol Reagent (Tiangen Biotech) according to the standard protocol. RNA was reverse-transcribed to synthesize cDNA using FastQuant RT Kit (Tiangen Biotech) according to the manufacturer’s protocol. The cDNA was used as template for quantitative PCR (qPCR) using SuperReal PreMix (Tiangen Biotech). qPCR was performed on an ABI 7500 system (Applied Biosystems). Gene expression was normalized to Gapdh and compared with control group. Primers used for analysis are listed in Supplementary Table S1. All samples were analyzed in triplicates and all experiments were repeated for three times.

### Gene silencing by siRNA or shRNA transfection

siGENOME Tet1 and Tet2 siRNAs and a non-targeting siRNA were purchased from Dharmacon. C2C12 cells were transfected with siRNAs using Lipofectamine 3000 (Invitrogen) according to the manufacturer’s protocol. Cells were cultured in 12-well plates and transfected with 30 pmol siRNA in complex with 10 μL of Lipofectamine 3000 dissolved in Opti-MEM solution (Gibco). After transfection for 6 h, the medium was replaced with fresh growth medium. Transfected cells were harvested 48 h later (for analysis of mRNA) or 72 h later (for analysis of protein). Each transfection experiment was repeated at least three times. To achieve persistent knockdown of Tet2, C2C12 cells was transfected with a Tet2 shRNA plasmid (shTet2) or a control scrambled shRNA plasmid (shCtrl) (Origene Technologies). Twenty-four hours after transfection, the cells were trypsinized, diluted and cultured in medium containing 2.5 μg/ml puromycin (Clontech) for 2 weeks for selection. Survived cells were used to subsequent experiments.

### Immunofluorescence staining

Cells grown on 0.01 mg/ml poly-L-lysine (Sigma)-coated coverslips were fixed with 4% paraformaldehyde for 15 min, washed twice with PBST (PBS + 0.5% Tween 20) and permeated with 0.5% Triton X-100 for 25 min. Cells were treated with 2 M HCl for 20 min and then blocked in 1% BSA, 0.1% Triton X-100 in PBS for 1 h at 37 °C. Cell samples were incubated with relevant primary antibodies against myosin heavy chains (MF20, Developmental Studies Hybridoma Bank; 1:100 dilution), TET2 (Abcam, ab94580; 1:100 dilution), 5-hydroxymethylcytosine (ActiveMotif; 39769; 1:400 dilution), 5-methylcytosine (Eurogentec; BI-MECY-0100; 1:600 dilution), for 1 h at 37 °C. After extensive washes with PBST, cells were incubated with Dylight 488 or Dylight 549 -conjugated anti-rabbit IgG (Abbkine, A23220/A23320;1:200 dilution) or anti-mouse IgG (Abbkine, A23210/A23310; 1:200 dilution) antibodies. Cells were counterstained with 5 μg/ml 4, 6-diamidino-2-phenylindole (DAPI) in PBS and then mounted onto the slides in antifading solution containing 0.25% DABCO. Images were acquired using an Olympus BX51 epifluorescence microscope. Fluorescence intensity was measured with Image-Pro Plus 6.0 Software by manually outlining each nucleus in a given field.

### Western blotting

To analyze the amount of Tet2 in cells, C2C12 cells at 0 and 6 d after differentiation induction were washed with PBS and lysed with lysis buffer (Beyotime). After being mixed with loading buffer (Applygen) and boiled for 10 min, denatured protein samples were separated by 8% SDS-PAGE gel and electrotransferred onto PVDF membranes. The membranes were blocked overnight at 4 °C in 5% milk in TBST buffer and then incubated for 2 h at room temperature with primary antibodies against TET2-specific (Abcam, ab94580; 1:500 dilution), myosin heavy chain (DSHB, MF20; 1:2000 dilution) or β-actin (Abcam, ab8227; 1:4000 dilution), followed by incubation with HRP-conjugated anti-rabbit IgG (Applygen; 1:3000 dilution) or anti-mouse IgG (Applygen; 1:4000 dilution) secondary antibodies for 2 h at room temperature. Immune complex were detected using Super ECL Kit (Applygen).

### DNA methylation analysis

Genomic DNA was extracted from C2C12 cells using the Genomic DNA Clean & Concentrator kit (Zymo Research). Bisulfite treatment and recovery of samples were carried out with the EZ DNA Methylation-Gold Kit (Zymo Research) according to the manufacturer’s instructions. Bisulfite sequencing primers were designed with the online MethPrimer software (Supplementary Table S1). PCR was performed with the Hot-Start Ex Taq DNA Polymerase (TAKARA), and the products were gel selected, purified using the TIANgel Midi Purification Kit (Tiangen Biotech) and cloned through TA cloning using the pEASY-T1 Simple Cloning Kit (TransGen Biotech). At least 10 clones were sequenced and the results were analyzed with BiQ Analyzer software.

### Statistical analysis

Statistical analyses were performed with GRAPHPAD PRISM 5 Software. Results are presented as means ± SEM. Data were analyzed by Student’s two-tailed t test. Differences were considered significant at three levels (*p < 0.05, **p < 0.01, ***p < 0.001).

## Additional Information

**How to cite this article:** Zhong, X. *et al*. Ten-Eleven Translocation-2 (Tet2) Is Involved in Myogenic Differentiation of Skeletal Myoblast Cells *in Vitro. Sci. Rep.* **7**, 43539; doi: 10.1038/srep43539 (2017).

**Publisher's note:** Springer Nature remains neutral with regard to jurisdictional claims in published maps and institutional affiliations.

## Supplementary Material

Supplementary Information
