# Supplementary material for: Ten-Eleven Translocation-2 (Tet2) Is Involved in Myogenic Differentiation of Skeletal Myoblast Cells in Vitro
Source: Sci Rep. 2017 Mar 8;7:43539. doi: 10.1038/srep43539 (PMC5341099; doi:10.1038/srep43539)
Supplement: Supplementary Information [file srep43539-s1.pdf]

# Supplementary information for: "Ten-Eleven Translocation-2 (Tet2) Is Involved in Myogenic Differentiation of Skeletal Myoblast Cells in Vitro"

Xia Zhong*, Qian-Qian Wang*, Jian-Wei Li, Yu-Mei Zhang, Xiao-Rong An, Jian Hou

State Key Laboratory of Agrobiotechnology and College of Biological Science, China Agricultural University, Beijing, China

* These authors contributed equally to this work

To whom correspondence should be addressed: Jian Hou, State Key Laboratory of Agrobiotechnology and College of Biological Science, China Agricultural University, Beijing 100193, China. Telephone: 86-10-62733355-16; E-mail: houjian@cau.edu.cn

**Supplementary Table S1.** Primer sets used in the quantitative RT-PCR and bisulfite sequencing（BSP）

| Gene name | Primer sequence | Amplicon size (bp) |
|---|---|---|
| **Tet1** | FP: 5’-TCATTCCAGACCGCAAGACC-3’<br>RP: 5’-TGACACCAGAGAAAGGACGC-3’ | 195 |
| **Tet2** | FP: 5’-CACCCATCCACACCCTTCACC-3’<br>RP:5’-TCTGGAGGCAGCTCCCATGAA-3’ | 174 |
| **Tet3** | FP: 5’-AGTTCCCTACCTGCGATTGTGT-3’<br>RP: 5’-CTCCTTGCCCGTGTAGATGACC-3’ | 173 |
| **Myf5** | FP: 5’-TCAGGAATGCCATCCGCTAC-3’<br>RP: 5’-AATCCAAGCTGGACACGGAG-3’ | 246 |
| **Myod** | FP: 5’-CGACACCGCCTACTACAGTG-3’<br>RP: 5’-GGTGGTGCATCTGCCAAAAG-3’ | 150 |
| **Myogenin** | FP: 5’-GCAATGCACTGGAGTTCGGTC-3’<br>RP: 5’-GTTGGGCATGGTTTCGTCT-3’ | 134 |
| **Myf6** | FP: 5’-CGGCTGGATCAGCAAGAGAA-3’<br>RP: 5’-CCACGTTTGCTCCTCCTTCC-3’ | 178 |
| **Myomaker** | FP: 5’-ACCATGTTCTTTGTGGCGTTC-3’<br>RP: 5’-GTTCATCAAAGTCGGCCAGT-3’ | 135 |
| **Gapdh** | FP: 5’-TGATGACATCAAGAAGGTGGTG-3’<br>RP: 5’-TCCTTGGAGGCCATGTAGGCCAT-3’ | 240 |
| **Myogenin-BSP** | FP: 5’-AGGAAAGAGAAGGTTAAGTGGATTT-3’<br>RP: 5’-ATTCCCCTTCCCTCTCCTTTT-3’ | 325 |
| **Myomaker-BSP** | FP: 5’-GGAGTGAATATTGAGGGTTGTATAG-3’<br>RP: 5’-AAATAAACTTCACCTTTTCCTAACC-3’ | 424 |
| **Myf6-BSP** | FP:5’-AGTTGCCTGGTTAGCAGGTTAGTGT-3’<br>RP:5’-CACCCAGATGGCATTTAATTAGTGC-3’ | 306 |
| **Dnmt1** | FP:5’-ACGGAAACCCAAGGAAGAGTC-3’<br>RP:5’-CAGCACCACTCTCTGTGTCTA-3’ | 107 |
| **Dnmt3a** | FP:5’- ACGGCAGAATAGCCAAGTTCA-3’<br>RP:5’- GACGTCTGTGTAGTGGACGG-3’ | 170 |
| **Dnmt3b** | FP:5’-TCTAACGTCAATCCTGCCCG-3’<br>RP:5’-CTCCTTGGGGCGGGTATAAT-3’ | 96 |

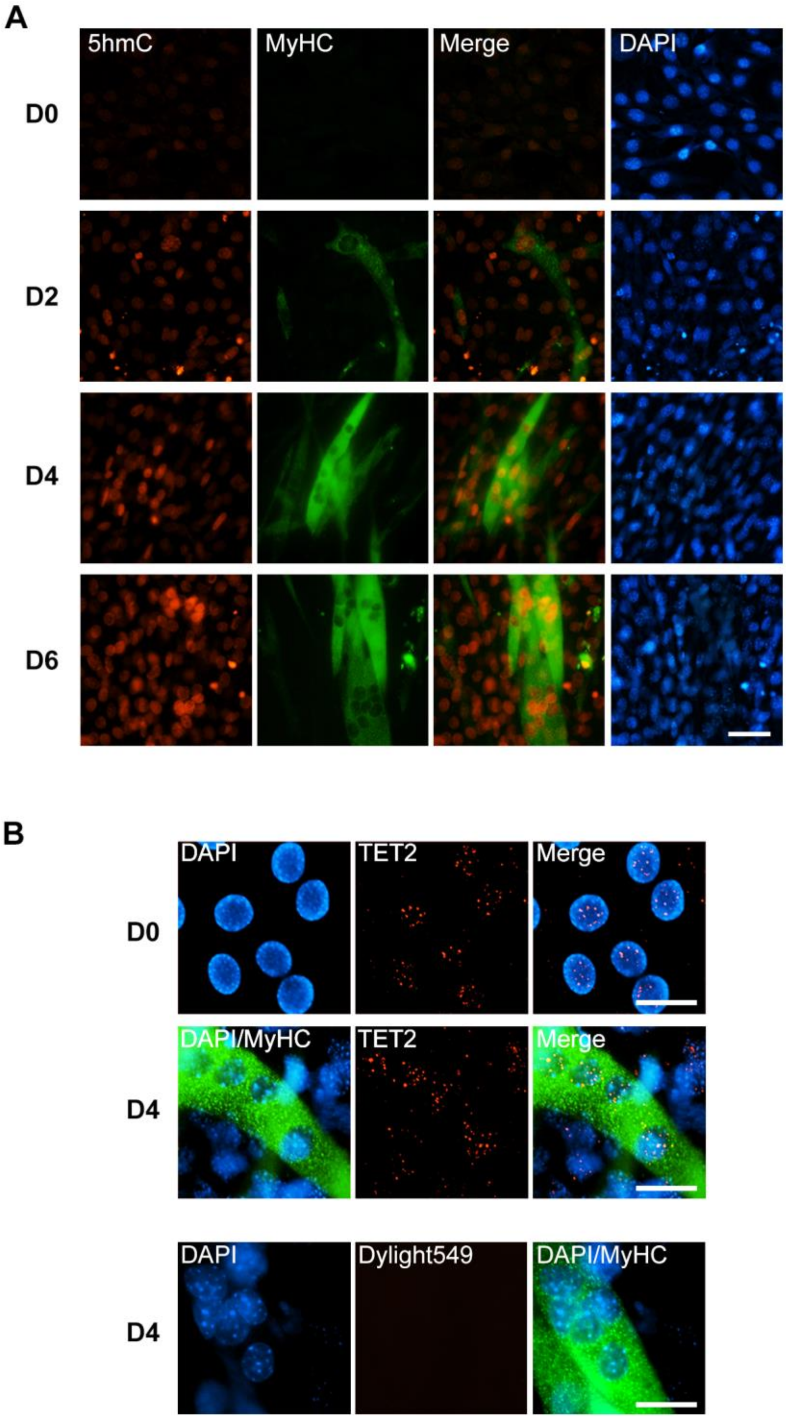


**Supplementary Figure S1.** (**A**) Immunofluorescence of 5hmC in nuclei during myoblast differentiation. 5hmC and MyHC were co-immunostained in C2C12 cells after differentiation induction for 0 to 6 d (D0-D6). D0 cells were essentially at myoblast stage because these cells were sampled just before shifted into differentiation medium. Nuclei were stained with DAPI. Scale bar, 50 μm. (**B**)

Localization of Tet2 protein in nuclei of myoblasts (D0) or myotubes (D4). Tet2 and MyHC were co-immunostained in C2C12 after differentiation induction for 4 d. Nuclei were stained with DAPI. Cells stained with only a secondary antibody (Dylight 549) serve as a negative control for 5hmC staining. Scale bar, 25 μm.

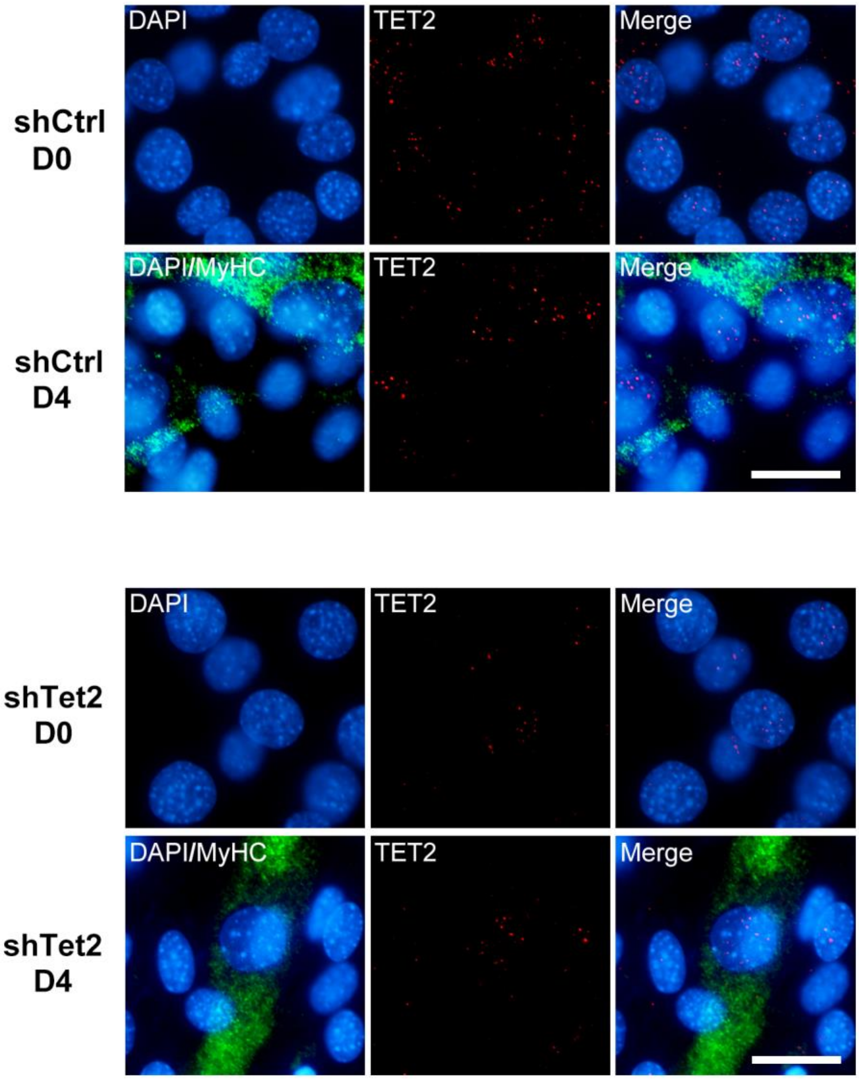


**Supplementary Figure S2.** Localization of Tet2 protein in Tet2-knockdown cells and control cells. C2C12 cells transfected with Tet2 shRNA plasmid (shTet2) or a control shRNA plasmid (shCtrl) were co-immunostained for Tet2 and MyHC at D0 or D4 after differentiation induction. Nuclei were stained with DAPI. Scale bar, 25 μm.

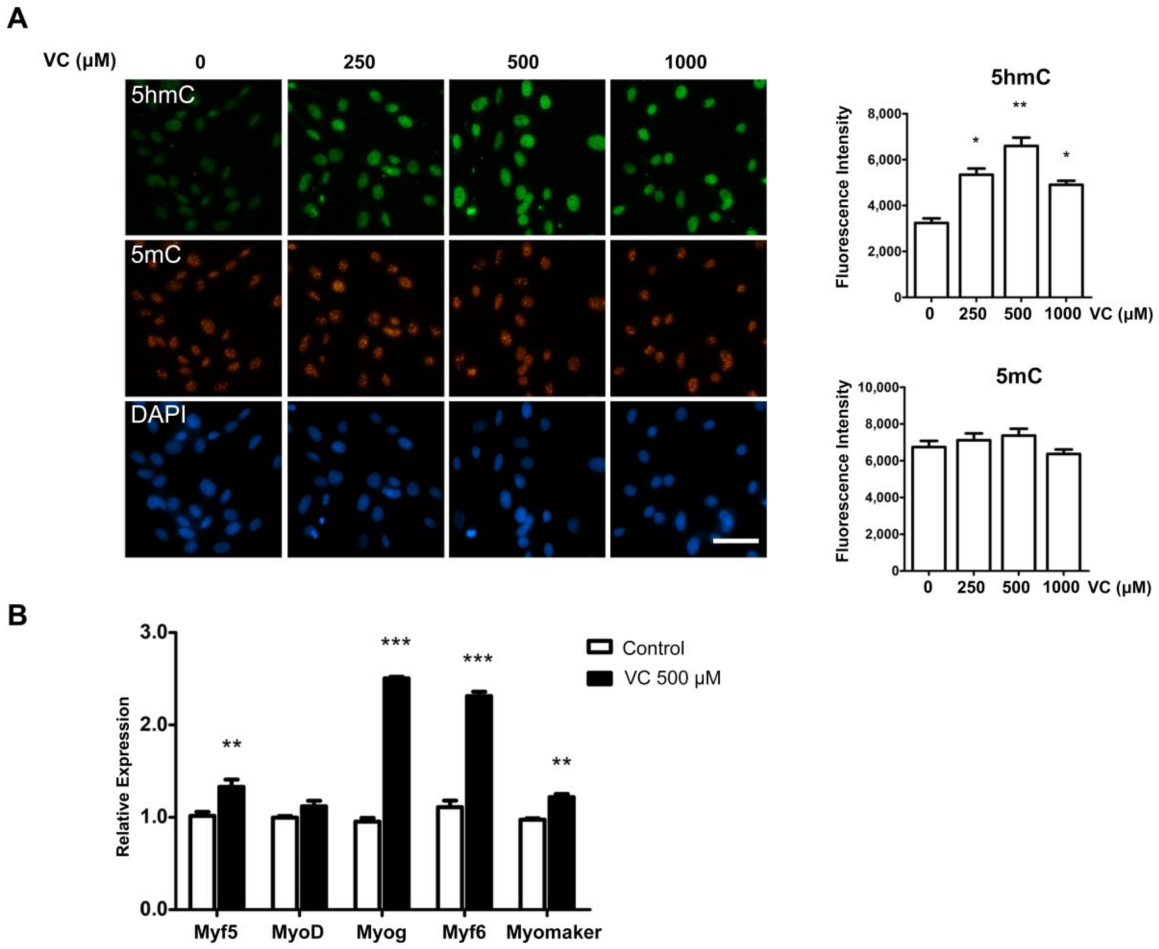


**Supplementary Figure S3. Vitamin C enhances 5hmC levels and promotes the expression of muscle-specific genes in C2C12 myoblast**. (**A**) *left row*, Immunostaining patterns of 5hmC and 5mC in C2C12 myoblast treated with different concentrations of vitamin C (VC; 0, 250, 500, 1000 μM). Nuclei were stained with DAPI. Scale bar, 100 μm. *right row*, Quantification analysis of 5hmC and 5mC immunofluorescence in VC-treated C2C12. Asterisks above columns represent significant differences among groups ($p < 0.05$). (**B**) qRT-PCR analysis for the expression of muscle-specific genes in C2C12 treated with 500 μM VC. Gapdh was used as an internal control. Data are presented as means ± SEM (n=3). *, $p < 0.05$; **, $p < 0.01$; ***, $p < 0.001$ as compared with C2C12 without VC treatment.

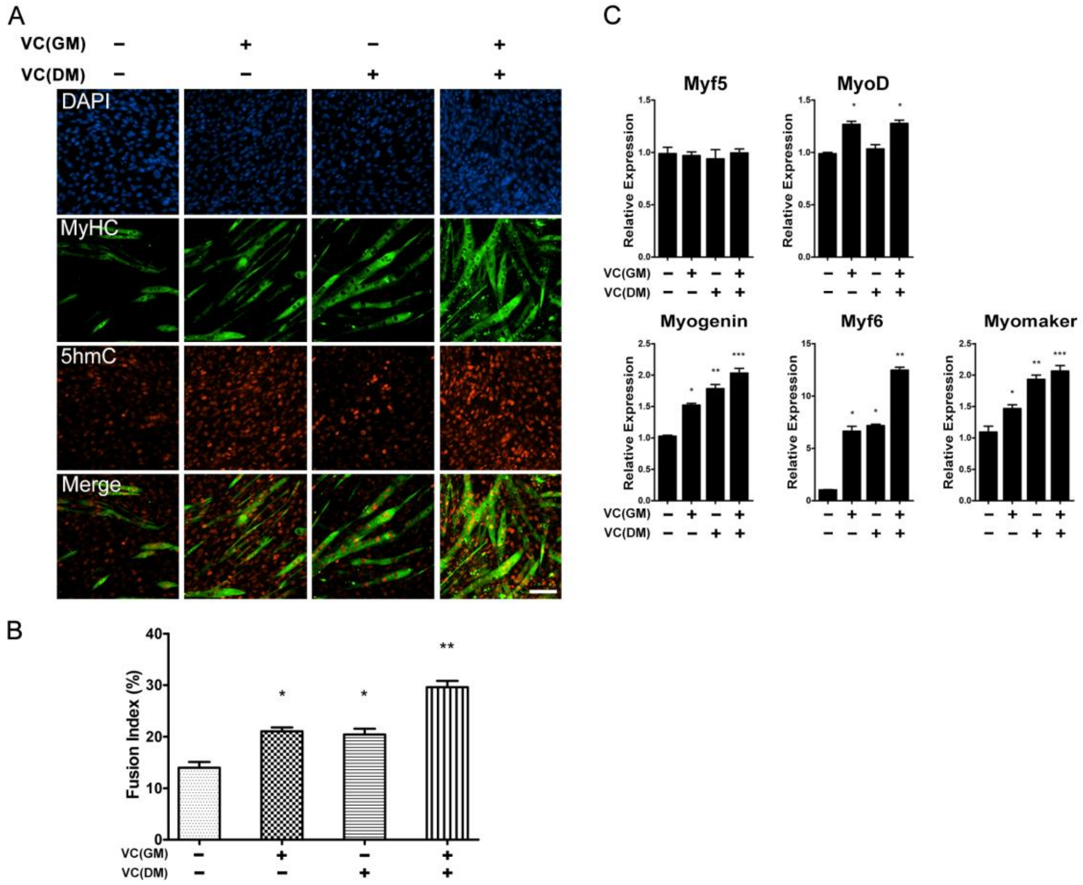


**Supplementary Figure S4. Vitamin C promotes myoblast differentiation and 5hmC formation during C2C12 differentiation**. C2C12 cells were cultured in growth medium (GM) with or without vitamin C (VC; 500 μM) for 48 h and then were transferred into differentiation medium (DM) with or without VC (500 μM). (**A**) Immunostaining patterns of 5hmC in C2C12 cells with different VC treatment. Cells after 4 d of differentiation were co-immunostained with anti-5hmC and anti-MyHC antibodies. Nuclei were stained with DAPI. Scale bar, 100 μm. Note that the addition of vitamin C in GM led to 5hmC increase in all cells including both differentiated and undifferentiated cells, while the addition of vitamin C in DM only increased the 5hmC level in differentiated cells. When vitamin C was added in both GM and DM, the 5hmC level was more increased but in both differentiated and undifferentiated cells. (**B**) Quantitative analysis for differentiation efficiency in different VC treatment groups. The fusion index was calculated as the ratio of the number of nuclei in MyHC-positive cells to the total number of nuclei present in the observation field.

Data are presented as means ± SEM (n=3). Asterisks above columns represent significant differences among groups ($p < 0.05$). (**C**) qRT-PCR analysis for the expression of muscle-specific genes in C2C12 after 4 d of differentiation induction. Gapdh was used as an internal control. Data are presented as means ± SEM (n=3). Asterisks above columns represent significant difference among the groups ($p < 0.05$).

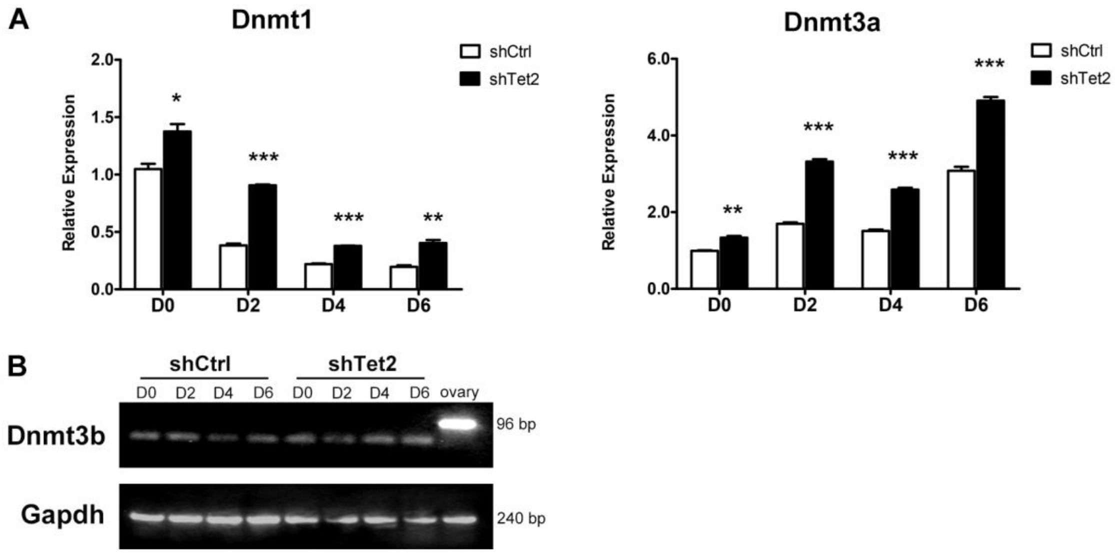


**Supplementary Figure S5**. Expression patterns of Dnmt family genes in Tet2 knockdown cells and control cells during myoblast differentiation. C2C12 cells transfected with Tet2 shRNA plasmid (shTet2) or a control shRNA plasmid (shCtrl) were induced to differentiate and mRNA levels of Dnmt family genes were analysed by qRT-PCR during differentiation from day 0 to day 6 (D0-D6). Gapdh was used as an internal control. Data are presented as means ± SEM (n=3). *, $p < 0.05$; **, $p < 0.01$; ***, $p < 0.001$. (**A**) qRT-PCR analysis for the expression of *Dnmt1* and *Dnmt3a*. *Dnmt1* transcripts are gradually decreased, while *Dnmt3a* expression is increased, during myoblast differentiation. Tet2 knockdown upregulates the expression of both *Dnmt1* and *Dnmt3a.* (**B**) Detection of *Dnmt3b* expression. No transcripts of *Dnmt3b* could be detected in both Tet2 knockdown cells and control cells. mRNAs extracted from mouse ovary serve as a positive control for Dnmt3b detection.

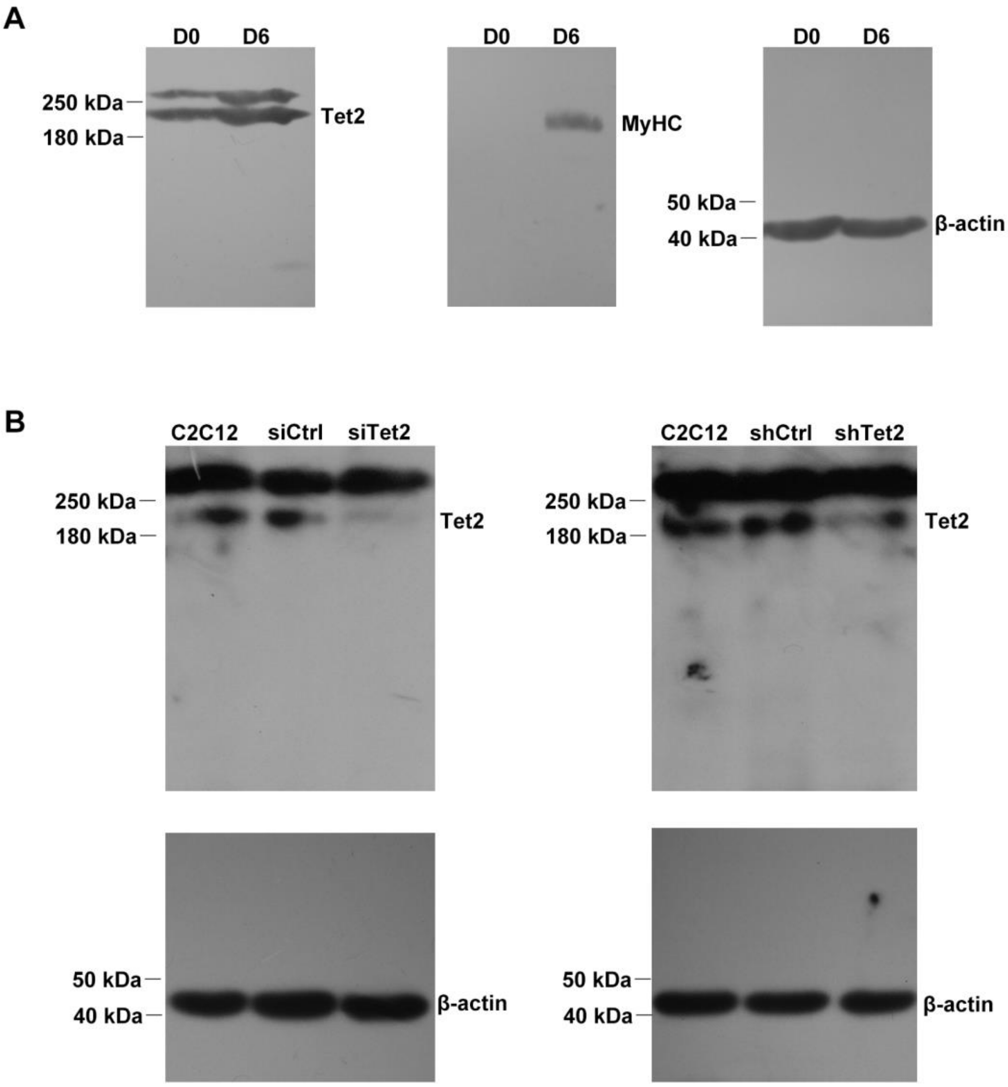


**Supplementary Figure S6**. Full-length western blots. (**A)** Blots related to Figure 1C. (**B)** Blots related to Figure 3B. While two band sizes for Tet2 blots are observed in full-length blots, the band of 224 kDa is the predicted Tet2. The identity of the additional larger band (approximately 300 kDa) is unknown. This is also explained in the product instruction provided by the antibody manufacturer (Abcam, ab94580).
